# Extended‐release buprenorphine treatment for opioid use disorder: A mixed‐methods study of response and experience

**DOI:** 10.1111/add.70106

**Published:** 2025-06-20

**Authors:** Natalie Lowry, Andrew McKechnie, Edward Day, Eilish Gilvarry, Fiona Cowden, Rachel Evans, Rosie Locke, Robbie Murray, Rob Vanderwaal, Stacey Johnstone, Zoe Hoare, Michael Kelleher, Luke Mitcheson, John Marsden

**Affiliations:** ^1^ Addictions Department School of Academic Psychiatry, Institute of Psychiatry, Psychology and Neuroscience, King's College London London UK; ^2^ Lambeth Drug and Alcohol Service South London and Maudsley NHS Foundation Trust London UK; ^3^ Dundee Drug and Alcohol Recovery Service Dundee Health and Social Care Partnership Scotland UK; ^4^ Solihull Integrated Addiction Service Birmingham and Solihull Mental Health NHS Foundation Trust Birmingham UK; ^5^ Institute for Mental Health, School of Psychology Birmingham University Birmingham; ^6^ Newcastle Treatment and Recovery Cumbria, Northumberland, Tyne and Wear NHS Foundation Newcastle Upon Tyne UK; ^7^ School of Health Sciences Bangor University Wales UK

**Keywords:** extended‐release buprenorphine, long‐acting injectable buprenorphine, mixed‐methods, opioid use disorder, patient experience, qualitative evaluation

## Abstract

**Background and aims:**

An investigation of 24 weeks of extended‐release buprenorphine (BUP‐XR; Sublocade®) treatment for adults with opioid use disorder (OUD). Study aims were to characterise variations in clinical response, investigate personal factors influencing BUP‐XR experience and identify opportunities to tailor treatment interventions.

**Design:**

A convergent parallel mixed‐methods evaluation embedded in a five‐centre, phase 3, randomised controlled trial of BUP‐XR versus daily oral methadone or sublingual buprenorphine.

**Setting:**

Four of five National Health Service addictions treatment clinics in England and Scotland from the trial.

**Participants:**

Participants were recruited after they completed the 24‐week endpoint. Forty‐nine participants (31%) from the trial completed the qualitative interview.

**Measurements:**

Three outcome measures from the trial's dataset were used descriptively: (1) fortnightly clinic visit administered TimeLine Follow‐Back interview and urine drug screen data on use of non‐medical opioids, cocaine and benzodiazepines; (2) the frequency version of the 11‐item Craving Experience Questionnaire administered at baseline and endpoint; and (3) the Structured Clinical Interview for DSM‐5 disorders for diagnosis of early OUD and cocaine use disorder (CUD) remission. Data visualisation (by heatmap) identified drug use response sub‐groups. A topic‐guided, semi‐structured qualitative interview was analysed by Interactive Categorisation.

**Findings:**

Three response sub‐groups were identified: Group 1 [14 (28.5%) of 49 participants] had the highest level of response, characterised by continuous abstinence from opioids, cocaine and benzodiazepines, improvements in craving control and mental and physical health in the majority, and a high level of remission and satisfaction with care; Group 2 [14 (28.5%) of 49 participants] had the next level of response, characterised by continuous abstinence from opioids, but some with opioid craving and some with compensatory use of cocaine and benzodiazepine to cope with anxiety and stress; Group 3 [21 (43.0%) of 49 participants] were not continuously abstinent from opioids during follow‐up, the majority had dual OUD and CUD at trial enrolment, some reported breakthrough opioid withdrawal symptoms during follow‐up, the majority reported improvements in mental health, but many reported opioid and cocaine cravings and compensatory use of cocaine and benzodiazepines.

**Conclusions:**

There appears to be variation in response and experience of extended‐release buprenorphine during the first six months of treatment, depending on substance use and physical health. This highlights the need for tailored treatment plans based on differing individual needs.

## INTRODUCTION

Daily oral liquid methadone (MET) and transmucosal tablet, film and lyophilizate wafer buprenorphine (BUP‐SL) are evidence‐based, standard‐of‐care maintenance pharmacotherapies for opioid use disorder (OUD) [1]. In England, between April 2022 and March 2023, 138 604 adults (≥18 years) were enrolled in MET or BUP‐SL in National Health Service (NHS) and third sector treatment clinics [2]. A further 70 282 adults were enrolled in MET or BUP‐SL with OUD and cocaine use disorder (CUD; typically, the alkaloid smokeable form) and 10 871 adults were enrolled in MET or BUP‐SL treatment with OUD and non‐medical benzodiazepine use disorder (BUD). CUD and BUD complicate MET and BUP‐SL care planning and predict poor clinical response and early discontinuation from treatment [3, 4].

In the last decade, two subcutaneous injectable, extended‐release formulations of buprenorphine (BUP‐XR) have been developed. In 2017, Indivior introduced a monthly product (Sublocade) in the United States (US) [5]. In 2018, Camurus introduced a weekly and monthly product (Buvidal) in Australia and Europe [6, 7]. In 2019, the Extended‐Release Pharmacotherapy for OUD study (EXPO) was initiated to determine if BUP‐XR was superior to MET or BUP‐SL. This was a multi‐site, randomised controlled trial (RCT) of 24 weeks of Sublocade (2 × 300 mg loading doses and 4 × 100 mg maintenance doses; *n* = 158) versus MET or BUP‐SL (standard‐of‐care doses; *n* = 156). EXPO was conducted in five NHS community clinics in England and Scotland: London (Brixton); North‐East (Newcastle); West‐Midlands (Solihull and Wolverhampton); North‐West (Manchester); and Tayside (Dundee) [8, 9]. The primary outcome measure was the number of days the participant abstained from non‐medical opioids from the second week of follow‐up to a 24‐week endpoint (range: 0–161 days).

Participants in the BUP‐XR group achieved more opioid abstinence (123 days vs. 104 days; *P* = 0.004). BUP‐XR was also associated with longer continuous opioid abstinence (95 days vs. 77 days; *P* = 0.005); reduced craving for opioids (various comparators; *P* < 0.05); longer treatment duration (145 days vs. 129 days; *P* = 0.029); and a higher probability of early OUD remission (probability of 0.75 vs. and 0.62; *P* = 0.042). There was no statistically significant treatment effect of BUP‐XR on the use of cocaine or benzodiazepines.

Qualitative research can achieve valuable insights into the treatment experiences of patients. A recent systematic review of the international qualitative literature on OUD pharmacotherapies identified that most patients initiate treatment to avoid the negative consequences of OUD and to attain a ‘normal life’ [10]. Early studies of BUP‐XR suggested that patients were motivated to avoid attending the community pharmacy for oral dosing and considered this a less stigmatising treatment option than MET or BUP‐SL [10]. Those with prior daily treatment experience valued a shift away from the routine of taking oral medicine [11, 12, 13]. However, there were also concerns that BUP‐XR dosing was not sufficiently adjusted to individual needs and the support needed during treatment was limited [14, 15].

Mixed‐methods designs are an extension of qualitative research. They combine qualitative and quantitative data to enrich, complement and extend the primary goal of a research study and its translational potential [16, 17, 18]. To our knowledge, there have been no mixed‐methods studies of BUP‐XR treatment reported to date. The EXPO trial embedded three mixed‐methods studies to investigate participants' clinical response and experience of 24 weeks of BUP‐XR treatment; longer‐term BUP‐XR treatment response and experience; and an comparative evaluation of BUP‐XR with a structured psychosocial intervention [19]. Here, we report the findings from the first of these three studies.

The aims of the study were: (1) to characterise participants' clinical response; (2) to investigate personal factors that influence BUP‐XR treatment experience; and (3) to identify opportunities to tailor interventions to meet individual needs.

## METHODS

This was a convergent parallel mixed‐methods study of the EXPO trial. The London‐Brighton and Sussex Research Ethics Committee (19/LO/0483) approved the protocol. The study was conducted at the Dundee, Solihull and Wolverhampton, Brixton and Newcastle study clinics among participants who were allocated to BUP‐XR and completed the 24‐week study follow‐up.

The protocol was reviewed by addiction clinic service user representatives, and the qualitative interview was piloted. We reviewed recommendations for sample size [20], and set a total target of 60 interviews. Consensus discussion among the research team determined if recruitment to the qualitative interview should be terminated if a saturation of experiences was achieved (i.e. no new experiences were reported).

### Qualitative interview

The Addiction Dimensions for Assessment and Personalised Treatment (ADAPT) informed the interview topic guide. The ADAPT is a clinical instrument developed for treatment care planning [21]. It includes 14 items that assess OUD severity; coincident health and social problems that are judged to be related to OUD as causes, effects, or are unrelated; and the person's strengths indexed by outlook, self‐management and social network support factors. The interview was broadly structured around BUP‐XR expectations (as all were naïve to this treatment) and their early and later experiences.

### Participant recruitment

Each participant allocated to BUP‐XR who attended their EXPO study 24‐week endpoint clinic visit was approached by the research team to determine their interest in completing a qualitative interview about their experiences while enrolled in the trial. Participants were compensated £20 for their time. N.L. and J.M. trained all interviewers. Informed, consenting volunteers completed a face‐to‐face, audio‐recorded interview with A.M., E.D., N.L., R.L., R.M., or S.J. for approximately 20 to 50 minutes in a private clinic room.

### Quantitative measures

Three outcome measures from the EXPO dataset were used:

1. Fortnightly clinic visit administered TimeLine Follow‐Back (TLFB) [22] calendar‐prompt interview, with immunoassay urine drug screen (UDS) procedure, to record daily use of non‐medical opioids, cocaine and benzodiazepines on each day of follow‐up, from the second week after randomisation (range: 0–161 days).
2. The frequency version of the 11‐item Craving Experience Questionnaire (CEQ‐F) [23] was administered at baseline and endpoint. The CEQ‐F recorded the person's rating of the frequency of intensity, imagery and intrusive aspects of craving for opioids and cocaine in the past week (each item was rated ‘not at all–constantly’; scored 0–10; total score: 0–110).
3. Structured Clinical Interview for Diagnostic and Statistical Manual of Mental Disorders, Fifth Edition (DSM‐5) disorders (SCID‐5‐RV) [24] for diagnosis of early OUD and CUD remission at endpoint.

### Data analysis and presentation

**FIGURE 1:**
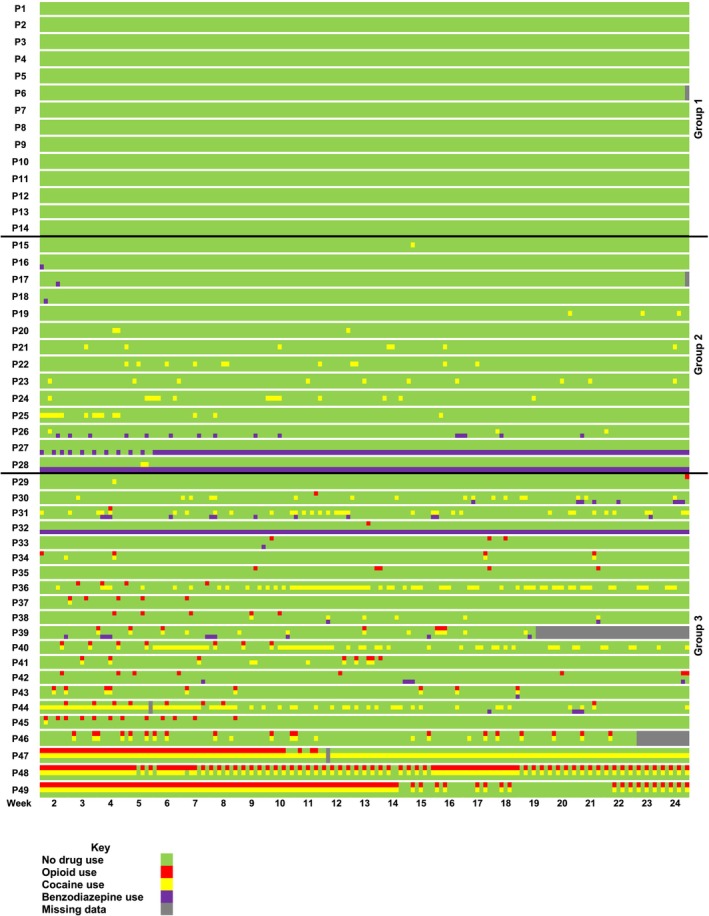
Heatmap data visualisation of abstinence, and use of opioids, cocaine and benzodiazepines during 24 weeks of study treatment with extended‐release buprenorphine (BUP‐XR). Group 1, continuous abstinence from opioids, cocaine and benzodiazepines; group 2, continuous abstinence from opioids; used cocaine or benzodiazepines or both drug on ≥1 days; group 3, used opioids on ≥1 days, and either abstained from cocaine and benzodiazepines or used one or both drugs on ≥1 days.

Quantitative and qualitative datasets were analysed separately [25]. The EXPO primary outcome measure (TLFB and UDS) was used to guide the convergent mixed‐methods analysis through the construction of a person‐level data visualisation (heatmap) of the use of opioids, cocaine and benzodiazepines (Figure 1). Each row of the heatmap represented a single participant. Each day was colour‐coded to indicate whether the participant was abstinent or they used one, two or three drugs. The heatmap was constructed by ranking the opioid use data from abstinence to regular use.

Interview audio recordings were transcribed verbatim. Participants were invited to review and comment on their transcripts before N.L. entered the data into NVivo 14 [26]. Qualitative data was analysed using iterative categorization [27, 28]. Each transcript was deductively coded using the ADAPT constructs, and residual data was inductively coded. Codes were merged into headings and subheadings, working toward a conceptual narrative. The Behavioural Model of Health Service Utilisation [29] was used to differentiate between individual and social determinants of BUP‐XR response. Coding was overseen by N.L. Two consensus meetings among authors were used to review identified concepts and themes. Exemplar quotes were accompanied by the participant's gender, study identification number and the baseline and week‐24 craving score.

## RESULTS

This study was conducted with 49 (31.0%) of the 158 participants allocated to BUP‐XR in the EXPO trial. The first and last participant was enrolled for the qualitative component of this study on 22 July 2020 and 31 January 2022. Enrolment by clinic was as follows: Brixton [15 (31.9%) of 47]; Newcastle [17 (32.7%) of 52]; Solihull and Wolverhampton [9 (39.1%) of 23]; and Dundee [8 (32.0%) of 25]. Brixton and Newcastle sites reached their recruitment target first. In the Solihull and Wolverhampton, and Dundee sites, participant recruitment was terminated when it was judged that a saturation of experiences was achieved.

The sample consisted of 39 males and 10 females (age range: 23–63 years) (Table 1). Nearly all participants received all scheduled doses of BUP‐XR [47 [95.9%] of 49]. Twenty‐six (53.1%) of 49 participants received all six injections per protocol (i.e. 2 × 300 mg loading doses and 4 × 100 mg maintenance doses). Twenty (40.8%) of 49 participants replaced one or more maintenance doses with a 300 mg dose. There were only minor differences in the demographic and clinical characteristics when compared with the main EXPO BUP‐XR sample, by age, ethnicity and remission status (OUD and CUD). Three drug use groups were identified (Table 2; Figure 1):

- Group 1 [14 (28.5%) of 49 participants] continuous abstinence from opioids, cocaine and benzodiazepines.
- Group 2 [14 (28.5%) of 49 participants] continuous abstinence from opioids; used cocaine or benzodiazepines or both drugs on ≥1 days.
- Group 3 [21 (43.0%) of 49 participants] used opioids on ≥1 days, and either abstained from cocaine and benzodiazepines or used one or both drugs on ≥1 days.

**TABLE 1:** Baseline characteristics of the sample and trial.

Characteristic	Sample (*n* = 49)	EXPO trial (*n* = 158)
Clinic—participants		
London	15 (30.6)	47 (29.8)
Newcastle	17 (34.7)	52 (32.9)
West Midlands	9 (18.4)	23 (14.6)
Tayside	8 (16.3)	25 (15.8)
Participant—demographic characteristics		
Age, y^a^	42 (9.5)	42 (7.9)
Sex, at birth		
Male	39 (79.6)	122 (77.2)
Female	10 (20.4)	36 (22.8)
Ethnicity		
White	42 (85.7)	136 (86.1)
Black	4 (8.2)	12 (7.6)
Mixed	2 (4.1)	7 (4.4)
Other	1 (2.0)	3 (1.9)
Clinical assessments		
DSM‐5 OUD status		
Severe	49 (100)	157 (99.4)
DSM‐5 CUD status		
Mild	2 (4.1)	10 (6.3)
Moderate	4 (8.2)	13 (8.2)
Severe	23 (46.9)	74 (46.8)
Drug use – past 28 days		
Opioids	23 (46.9)	64 (40.5)
Cocaine	19 (38.8)	73 (46.2)
Benzodiazepines	8 (16.3)	34 (21.5)
Craving for opioids^a^		
CEQ‐F	22 (30.6)	28 (33.1)
Craving for cocaine^a^		
CEQ‐F	21 (32.0)	27 (34.2)

Data are *n* (%).

BUP‐XR, extended‐release buprenorphine; CEQ‐F, Craving Experience Questionnaire ‐ frequency version; CUD, cocaine use disorder; DSM‐5, Diagnostic and Statistical Manual of Mental Disorders, Fifth Edition; OUD, opioid use disorder.

^a^ Mean (SD).

**TABLE 2:** OUD remission, drug use and endpoint (week‐24) opioid and cocaine craving by group.

Outcome	Group 1 (*n* = 14)	Group 2 (*n* = 14)	Group 3 (*n* = 21)	Overall (*n* = 49)
Early remission				
OUD	14 (100)	13 (92.9)	15 (71.4)	42 (85.7)
CUD	9 (64.2)	5 (35.7)	5 (23.8)	19 (38.8)
Drug use at baseline—past 28 days				
Opioids	3 (21.4)	4 (28.6)	15 (71.4)	22 (44.9)
Cocaine	2 (14.3)	6 (43.9)	11 (52.4)	19 (38.8)
Benzodiazepines	0 (0)	3 (21.4)	5 (23.8)	8 (16.3)
Drug use during follow‐up—past 161 days				
Opioids				
Continuously abstinent	14 (100)	14 (100)	0 (0)	28 (57.1)
Use on ≥1 days; range	0 (0)	0 (0)	21 (100); 1–108	21 (42.9); 1–108
Cocaine				
Continuously abstinent	14 (100)	3 (21.4)	3 (14.3)	20 (40.8)
Use on ≥1 days; range	0 (0)	11 (78.6); 1–15	18 (85.7); 1–160	29 (59.2); 1–160
Benzodiazepines				
Continuously abstinent	14 (100)	8 (57.1)	11 (52.4)	33 (67.3)
Use on ≥1 days; range	0 (0)	6 (42.9); 1–161	10 (47.6); 1–161	16 (32.7); 1–161
Craving at baseline—past 7 days				
CEQ‐F – opioids^a^	12 (25.0)	10 (22.0)	39 (32.8)	22 (30.6)
CEQ‐F – cocaine^a^	7 (22.9)	20 (32.3)	33 (34.4)	21 (32.0)
Craving at endpoint—past 7 days				
Opioids				
CEQ‐F score ≥1	0 (0)	3 (21.4)	8 (38.1)	11 (22.4)
CEQ‐F mean (SD); range^b^	0 (0); 0–0	11 (5.6); 6–17	32 (30.9); 2–98	26 (27.7); 2–98
Cocaine				
CEQ‐F score ≥1	1 (7.1)	4 (28.6)	13 (61.9)	18 (36.7)
CEQ‐F mean (SD); range^b^	4 (0); 4–4	43.8 (27.9); 15–82	37 (29.4); 2–96	35 (28.8); 2–96

Data are *n* (%).

CEQ‐F, Craving Experience Questionnaire‐frequency version; CUD, cocaine use disorder; OUD, opioid use disorder.

^a^ Mean (SD).

^b^ Mean among those with a score of 1 or more.

Two major themes and 12 sub‐themes were identified from the analysis of the interview transcripts: (1) individual factors associated with treatment response (substance‐related thoughts and behaviours, cravings, withdrawal symptoms, dosing, physical health and mental health); and (2) Social factors associated with treatment response. Given space limitations in this report, findings on social factors are reported elsewhere.

### Group 1: Continuous abstinence from opioids, cocaine and benzodiazepines (*n* = 14)

The clinical disorder of the first group was predominantly OUD, with the majority responding to daily treatment before allocation to BUP‐XR. Two members of the group used cocaine at baseline and desisted. There was no baseline or compensatory benzodiazepine use. The group was characterised by the highest level of clinical response and early remission. Each member was continuously abstinent from opioids and did not report craving. The majority [8 (57.1%) of 14] reported improvements in mental health. Two (14.3%) participants reported breakthrough opioid withdrawal symptoms, and they described how they received a ‘rescue’ dose of BUP‐SL or received 300 mg of BUP‐XR at the next dosing visit. These participants were appreciative of the medical response they received:

‘I contacted her [the clinic doctor], explained to her everything what was happening, that I was getting the shakes, cold sweats, hot sweats, doubled up; she says—right we'll get something sorted out for you, we'll get you a top up [BUP‐SL]—and she did.’ (M, P6, CEQ‐F baseline‐endpoint: 0–0 for opioids; 0–0 for cocaine)

Four (28.6%) of 14 participants disclosed their history of chronic pain before enrolling in the EXPO trial. Two considered that BUP‐XR had helped in reducing their pain. The other two reported that although they were still suffering, they were coping better. One noted:

‘I am in constant pain, every night before I go to bed. I just said to myself, take your time as you know you have got this illness you are dealing with. And that is sort of my mindset right now.’ (M, P14, CEQ‐F baseline‐endpoint: 0–0 for opioids; 0–0 for cocaine)

The remaining four participants reported wanting heroin on some occasions, but these were fleeting and were not distressing experiences. There were also reports of confidence in managing brief urges through reframing thoughts. During the first few weeks of BUP‐XR, one participant recalled episodic and procedural craving‐related memories about heroin that they were able to cope with:

‘[I was] not thinking about [heroin] itself, but thinking about how to go and get it, the people that I was around, the places that I would go…I just wanted to be there, around it. So I just started replacing it with other thoughts and soon enough it kind of faded away.’ (F, P13, CEQ‐F baseline‐endpoint: 86–0 for opioids; 86–4 for cocaine)

Several participants reported experiencing distressing panic‐type sensations in reaction to the topic of heroin and crack cocaine during conversation:

‘[BUP‐XR] changes something, the way you think, or just completely blocks it; but even if I think about it [heroin and crack cocaine] I feel sick; if someone talks about it…it makes everything in my stomach and chest, makes me feel like I'm going to throw up…I just remember the feeling from it, and the taste and everything, it's horrible.’ (M, P2, CEQ‐F baseline‐endpoint: 0–0 for opioids; 0–0 for cocaine)

The participant went on to say that they had later reflected on these experiences and now felt they could cope better by reminding themselves that the BUP‐XR was working as intended and recalled thoughts of gratitude about the opportunity they had in the study to receive a new treatment in the service of their recovery. Another participant found that being on BUP‐XR made them feel normal and reflected on the reactions to the short half‐life of daily BUP‐SL:

‘You don't get any of those emotional ups and downs anymore that you get with [BUP‐SL], so many peaks and troughs in your…in your sort of emotions…and it makes it difficult to balance that, whereas having it injected once a month, I suppose it keeps the chemicals in balance and your natural systems can just flow normally. I've felt better than what I felt for probably 20 years. (M, P5, CEQ‐F baseline‐endpoint: 36–0 for opioids; 4–0 for cocaine)

In another description of a return to normality, a participant reported a profound change in their thinking and sense of self:

‘My mind is crystal clear; before it was kind of clouded…you are looking into a lake or pond and it's green, or brown and cloudy, yeah and you cannot see the bottom. That's what it was like with [BUP‐SL], but soon as I went on to taking this [BUP‐XR], within days, a week, it was like looking, I could see the bottom. I could see all the fish, crystal clear and I knew, I had a direction, a purpose, it give me a drive, and I felt normal, I felt back to who I was when I knew who I was kind of thing. I had no cloudy cotton wool brain or anything. I was, I was normal as everybody else in society.’ (M, P4, CEQ‐F baseline‐endpoint: 0–0 for opioids; 0–0 for cocaine)

### Group 2: Continuous abstinence from opioids; use of cocaine or benzodiazepines or both (*n* = 14)

In contrast to the first group, group 2 had more members with dual OUD‐CUD at baseline, with three using benzodiazepines. All achieved continuous opioid abstinence during follow‐up, but three (21.4%) of 14 participants had mild opioid cravings at the endpoint. There was a mixed response for those using cocaine and benzodiazepines at baseline. The majority reported that BUP‐XR was effective at maintaining their opioid tolerance. Two (14.3%) of 14 reported mild withdrawal symptoms after the first loading dose. One recalled a brief and resolving phase of discomfort when they started treatment:

‘When I transitioned over from [BUP‐SL] to injections, there was a few days where my body was getting used to it, but I wouldn't say anything that was really bad; it was just more of my body getting used to the different way of being administered into my body really—felt a bit fatigued, it only lasted like 48 hours’. (M, P18, CEQ‐F baseline‐endpoint: 0–6 for opioids; 0–0 for cocaine)

An appreciation of the benefits of a longer BUP effect, coupled with the ability to step away from the community pharmacy, gave rise to several positive reflections. One participant described how this had enabled them to have time to reflect on their life:

‘Because [BUP‐XR] lasts so long inside you, you've got more time to, to get your head around, of not going back on to it [drug use].’ (M, P23, CEQ‐F baseline‐endpoint: 22–0 for opioids; 32–5 for cocaine)

There was an indication of compensatory cocaine use within this group, as only six participants reported cocaine use at baseline, but 11 (78.6%) of 14 participants used cocaine on 1 to 15 days, and 4 (28.6%) of 14 reported cocaine cravings during the follow‐up period. Motivations varied, but coping with stress was common. One participant reported starting cocaine use in the last month of follow‐up as a means of coping with a relationship breakdown:

‘I've went through a breakup, and I've started using a little bit of cocaine again, not anywhere near to the extent as that I was. But if I don't monitor it quickly, it can spiral out of control to where it was. It causes an argument with my wife, and I react by going out and taking hard drugs.’ (M, P19, CEQ‐F baseline‐endpoint: 0–0 for opioids; 66–82 for cocaine)

Two (14.3%) of 14 participants said that they did not experience cravings, but did use cocaine when around other people:

‘I can sit with people who are sitting smoking it [cocaine] you know where I used to before as I used to injected it, but I can sit with people and they're like smoking away…I have tried a couple of lines [cocaine] and it's not done anything, not a thing to me being on the injection, so I feel you know it's a waste of time.’ (F, P28, CEQ‐F baseline‐endpoint: 0–0 for opioids; 10–0 for cocaine)

One participant reported opioid cravings when using cocaine, because they did not like the feeling of being ‘too high’ from cocaine. They were no longer able to use opioids to ‘come down’ from cocaine, given the opioid blockade of BUP‐XR:

‘[Before the study] If I had coke or crack and I would have a bit of brown [heroin] to come down…I know there is no point in using [heroin] so I didn't bother, I knew I couldn't, so I just had to sit feeling uncomfortable [after using cocaine].’ (M, P22, CEQ‐F baseline‐endpoint: 80–0 for opioids; 80–15 for cocaine)

There was an increase from three to six (42.9%) of 14 participants using benzodiazepines, with two participants used most days. One participant took benzodiazepines to help them sleep:

‘If I feel like I've not had a good night's sleep, well, for maybe four or five days, I start to feel my head overthinking and that and I go to bed and try to sleep and I'll maybe take two or three of the street Valium and that's about it…and my sleep pattern seems to be a lot better just now.’ (M, P26, CEQ‐F baseline‐endpoint: 4–0 for opioids; 0–0 for cocaine)

Another participant described sustained benzodiazepine use to manage their anxiety:

‘I went away and took a couple [Valium] when I start worrying about my girlfriend. She makes my anxiety go through the roof.’ (M, P27, CEQ‐F baseline‐endpoint: 0–0 for opioids; 0–0 for cocaine)

Another participant reported trying multiple compensatory substances, as their primary substance was opioids, and they were no longer able to feel the effects:

‘That sort of cross‐addiction did go over to the other medications that I had missed. I started to use pregabalin more and illicit drugs…I tried methamphetamine, Adderall so it did switch my mind to think what other things I can try now…I am covered with opiates what else can I use?’ (M, P24, CEQ‐F baseline‐endpoint: 0–17 for opioids; 0–40 for cocaine)

This group also reported improvements in their mental health and generally no physical health issues. One participant found that they were starting to be able to differentiate between withdrawal symptoms and potential menopause symptoms that had previously been misattributed:

‘I can't get rid of is the sweats I don't know if it's the menopause or if it's the side effects of these, I get flushes, my mum is like that I took the menopause in my early forties, for god's sake I'm forty‐five.’ (F, P28, CEQ‐F baseline‐endpoint: 0–0 for opioids; 10–0 for cocaine)

### Group 3: Use of opioids on ≥1 day; abstinence from cocaine and benzodiazepines or use of one or both drugs on ≥1 day (*n* = 21)

In contrast to groups 1 and 2, the majority of the third group had OUD‐CUD at baseline. Five (23.8%) of 21 were using benzodiazepines, and relatively high levels of opioid craving were reported. Continuous opioid abstinence was not attained in this group—although there was a very wide range of opioid use from 1 day to most of the follow‐up. Seven individuals in this group experienced withdrawal symptoms. One said:

‘On the lower dose, the 100 [mg], I've experienced the sort of, you get this sneezing where it is like repeated sneezing over and over.’ (M, P42, CEQ‐F: 17–35 for opioids; 0–0 for cocaine)

Another commented on the later phase of the monthly dosing interval:

‘Three weeks into it, starting to feel a wee bit unwell.’ (M, P32, CEQ‐F baseline‐endpoint: 0–0 for opioids; 0–4 for cocaine)

Another said that they could feel the effects of heroin in the final days before their next injection:

*‘*I think it was maybe my subconscious thinking; once I did it [used heroin] maybe two days before my next injection and that time I think I felt it, actually, felt a bit the effect of heroin but not like it was as strong as normally.’ (F, P49, CEQ‐F baseline‐endpoint: 57–41 for opioids; 65–43 for cocaine)

One participant in this group experienced pre‐existing ongoing pain, with symptoms worsening because of no longer being disguised by substance use:

‘My body pains been better, obviously some of my severe injuries like with my kneecaps hurt, parts of me better, some parts I feel [the pain] more now because it has stopped being masked with the drug.’ (M, P34, CEQ‐F baseline‐endpoint: 13–6 for opioids; 2–14 for cocaine)

Eighteen (62.1%) of the 29 participants reported improvements in mental health. One participant reported an increased ability to cope with situations because of having more mental space that was previously occupied by substance use:

‘I'm starting to learn how to deal with certain situations and certain things in life that before I'd bury my head in drugs and not worry about it. Now I'm more mentally aware of what's going on and I now have to try and find new ways of learning, coping with certain situations that I wouldn't have had to dealt with before.’ (M, P34, CEQ‐F baseline‐endpoint: 13–6 for opioids; 2–14 for cocaine)

Four (19.0%) of 21 participants described no longer being able to feel the effects of heroin. Three participants reported compensatory cannabis or cocaine use to occupy time previously spent using opioids. Whereas one participant used alcohol and cigarettes to facilitate the ‘come down’ from cocaine:

‘I would smoke more cigarettes, maybe I would have some alcohol…I would have a glass of wine or whatever or maybe some spirit, but because even with I'm not doing that much crack that I would be so crazy up that I could not even focus.’ (F, P49, CEQ‐F baseline‐endpoint: 57–41 for opioids; 65–43 for cocaine)

Many participants in the third group experienced opioid and cocaine cravings: eight (38.1%) of 21 and 13 (61.9%) of 21, respectively. One participant described having daily sensory cravings:

‘They never go away … it's like a voice in your head saying get some, I need some, I need this, I need that. You know if you are like around someone who is using and you get the smell and you crave the taste, you crave the sight of it … I get them [cravings] every day.’ (M, P41, CEQ‐F baseline‐endpoint: 48–98 for opioids; 96–96 for cocaine)

Another found that they were craving cocaine as they viewed it as a forbidden fruit:

‘I didn't know what to do with myself, and I think maybe that was another reason as well as sort of the forbidden fruit thing and wanting to do something naughty to fill my time with, switching from heroin to cocaine, the fact that I didn't have nee, like I say nee structure I didn't, kind of wanted something to occupy the time.’ (M, P30, CEQ‐F baseline‐endpoint: 59–26 for opioids; 12–73 for cocaine)

## DISCUSSION

This mixed‐methods study showed that 28 (57.1%) of 49 participants who completed 24 weeks of study follow‐up (32%–39% of the enrolled samples in the EXPO trial in the four clinics) achieved continuous opioid abstinence for 161 days (the EXPO endpoint).

Forty‐seven (95.9%) of 49 participants received all six scheduled BUP‐XR injections. Their efficacy response aligns with the effect from the EXPO study for participants who received BUP‐XR as planned [adjusted incident rate ratio 1.21; 95% CI = 1.04–1.41; *P* = 0.02; *n* = 286 (EXPO)] and exceeds the treatment effect reported by clinical trials in Australia and the US (38.1% and 35.1%, respectively) [30, 31]. Data visualisation of the participants' opioid, cocaine and benzodiazepine use, revealed three distinct and non‐overlapping treatment response groups. The first was predominantly a primary OUD group, who were enrolled in the study after a good response to daily treatment, and BUP‐XR proved to be highly successful. All were continuously abstinent from opioids, cocaine and benzodiazepines, nearly all were in early OUD remission. The second group had members with OUD‐CUD at baseline, and although they achieved continuous abstinence from opioids, there was isolated to frequent compensatory cocaine and benzodiazepine use. The third group were the most complex. The majority had OUD‐CUD at baseline. None attained continuous opioid abstinence, and there were many reports of intrusive and distressing cravings. The heatmap for this group displayed drug use patterns that varied widely. One participant abstained continuously until the penultimate day of follow‐up, and they abstained from cocaine apart from 1 day. Another three also used opioids on a single day, but with more cocaine and benzodiazepine usage. The remaining participants appeared to have a repeating cycle of drug use, with three using opioids and cocaine for most follow‐up days.

Across all groups, many participants reported improvements in their mental health. As previously stated, BUP‐XR can alleviate some of the challenges of living day‐to‐day [11, 12, 13] and improve life satisfaction [32]. Our participants found that the monthly regime allowed for more time to comprehend a life that is abstinent from substances and relieved them from the daily physical and emotional fluctuations from receiving MET or BUP‐SL. This may reflect the quantitative findings from EXPO—longer abstinent days, longer continuous abstinence and longer time enrolled in BUP‐XR treatment [8].

Reflecting on how to tailor BUP‐XR to address individual needs, we recorded no recommendations from members of group 1 on how ongoing relapse prevention support could be improved. In our view, the transition to BUP‐XR should not be accompanied by a reduction in clinical contact time to align with clinic visits for dosing. Regular keyworker support is indicated to build and maintain the patient's response to this new regimen. Taking an interest in how patients are spending their time; how they appraise and benefit from craving‐free time; and checking on areas of individual medical and social care needs are all key elements of personalised care.

In groups 2 and 3, reports of cocaine or benzodiazepine were typically motivated by coping with stress, anxiety or sleep difficulties and could benefit from a psychosocial intervention focusing on cognitive and affective factors [33]. Physical health for others was a concern, and one participant reported difficulties differentiating between menopause symptoms and withdrawal symptoms, which is common for those with OUD [34]. Others reported the surfacing of negative emotions and preoccupation with chronic pain. BUP‐XR is approved for the treatment of OUD, but BUP itself has been used effectively for chronic pain management [35]. For chronic pain, the lower risk of respiratory depression with BUP compared with a full agonist and a longer half‐life may confer several advantages for patients with chronic pain, but there is a need for risk management and careful monitoring [36, 37]. In addition to pharmacological treatments for menopause and chronic pain, psychological interventions such as cognitive behavioural therapy are also recommended [38, 39].

Additionally, many expressed cue‐elicited cravings for opioids from using crack cocaine, for example, using heroin to mitigate the sensations felt with crack cocaine use. However, because of perceived or actual blockade effects of BUP‐XR, the mitigating sensations were no longer effective, therefore, in some instances, this behaviour was negatively reinforced. We have developed a cognitive therapy to help patients cope and re‐consolidate experiences like this [40, 41], which could be an appropriate time in the patient's treatment journey to offer this psychotherapy. Additionally, contingency management with MET or BUP‐SL has been associated with longer periods of abstinence for those with CUD‐OUD [42].

It appeared self‐evident that group 3 did not achieve the benefit from treatment that they were seeking. As has been reported in previous studies, some individuals tested the BUP‐XR opioid blockade [14]. In the present study, some reported being able to feel opioid effects without the negative consequences (i.e. withdrawal symptoms), which may have led to sustained opioid use. However, when group 1 experienced suboptimal BUP‐XR dosing, they were more likely to advocate for themselves to healthcare professionals. The same collaboration was not present for the other groups despite the clinic team offering a dose increase. Conversely, these findings suggest the need for a more collaborative treatment and assessment of suboptimal BUP‐XR dosing, which can be altered for each patient to accommodate individual needs.

### Strength and limitations

It is sometimes unclear how participant quotes in qualitative studies are selected and this may point to a risk of confirmation bias [31, 32]. We think a key strength of our study is that we used drug use responses to identify distinct sub‐groups. Exemplar quotes for each group had a clear context to illustrate experiences during follow‐up, that inform ways to build on early success and address ongoing needs. These are exploratory findings, but they are part of an effort to investigate OUD clinical phenotypes and risk profiling [43, 44].

The findings from the study should be considered in the context of several limitations. We recruited 31% of the EXPO participants allocated to BUP‐XR at the endpoint, although there was a very high level of adherence, with 95.9% receiving all six injections, our findings are not reflective of all trial participants. A ‘real world’ study in the United States found that there is commonly a ‘honeymoon effect’ for early adopters of BUP‐XR treatment, however, there are higher longer‐term dropout rates than BUP‐SL [45]. Therefore, it would be beneficial to conduct longer‐term mixed‐methods research on BUP‐XR, including participants who discontinued BUP‐XR treatment, to understand this phenomenon. It is also important to note that most of this sample had previous experience with medications for OUD, and it would be beneficial for future research to have views from people who have no prior experience with other medications for OUD.

## CONCLUSIONS

In a mixed‐methods study of 49 participants in a clinical trial who completed 24 weeks of BUP‐XR for OUD, the majority of study participants achieved continuous abstinence from opioids—the primary purpose of BUP‐XR. Because of the less frequent prescribing of BUP‐XR than MET or BUP‐SL, keyworker time can be redirected to focus on maximising early treatment gains and assessing for additional support needs. A stepped treatment approach could be used for those who continue drug use, by offering psychological interventions to cope with managing sleep, stress or physical health conditions.

## AUTHOR CONTRIBUTIONS

**Natalie Lowry:** Conceptualization (lead); data curation (lead); formal analysis (lead); methodology (lead); project administration (lead); resources (lead); supervision (lead); writing – original draft (lead); writing – review and editing (lead). **Andrew McKechnie:** Data curation (equal); writing – review and editing (supporting). **Edward Day:** Conceptualization (supporting); data curation (equal); writing – review and editing (supporting). **Eilish Gilvarry:** Conceptualization (supporting); writing – review and editing (supporting). **Fiona Cowden:** Conceptualization (supporting); writing – review and editing (supporting). **Rachel Evans:** Data curation (supporting); writing – review and editing (supporting). **Rosie Locke:** Data curation (equal); writing – review and editing (supporting). **Robbie Murray:** Data curation (equal); writing – review and editing (supporting). **Rob Vanderwaal:** Writing – review and editing (supporting). **Stacey Johnstone:** Data curation (equal); writing – review and editing (supporting). **Zoe Hoare:** Data curation (supporting); writing – review and editing (supporting). **Michael Kelleher:** Conceptualization (supporting); writing – review and editing (supporting). **Luke Mitcheson:** Conceptualization (supporting); supervision; writing – review and editing (supporting). **John Marsden:** Conceptualization (lead); formal analysis (equal); funding acquisition (lead); methodology (equal); supervision (lead); writing – original draft (equal); writing – review and editing (equal).

## DECLARATION OF INTERESTS

F.C. and E.G. declare collaborative research grant funding for the EXPO study from Indivior [study sponsor: King's College London (KCL) and South London and Maudsley NHS Trust (SLaM)]. In the past 3years, E.D. has declared research grant funding from the National Institute for Health Research [NIHR; trial of behavioural reinforcement of acamprosate for alcohol use disorder (AUD); study sponsor: KCL and SLaM]; and Indivior for the EXPO study. E.D. is the United Kingdom (UK) Government National Recovery Champion, seconded part‐time to the Home Office. In the past 3 years, M.K. declares research grant funding from Indivior for the EXPO study, and from Beckley PsyTech for a phase 2 trial of 5‐MeO‐DMT for AUD (sponsor: Beckley PsyTech). M.K is the national clinical advisor for the Office for Health Improvement and Disparities, English Department of Health and Social Care. In the past 3 years, L.M. declares research grant funding from the NIHR for a realist evaluation of services for people with co‐occurring mental health and substance use and a study of ketamine for AUD; from Indivior for the EXPO study; and from Beckley PsyTech for a phase 2a trial of 5‐MeO‐DMT for AUD. L.M. has a clinical psychology secondment at the Office for Health Improvement and Disparities, English Department of Health and Social Care. In the past 3 years, J.M. declares research grant funding from the NIHR for a trial of behavioural reinforcement for AUD medication; from Indivior for the EXPO study; from Indivior for the EXPO study; and from Beckley PsyTech (phase 2a trial of 5‐MeO‐DMT for AUD). He is the senior scientific advisor for the Office for Health Improvement and Disparities, English Department of Health and Social Care and a clinical academic consultant for the US National Institute on Drug Abuse, Clinic for Clinical Trials Network. J.M. declares honoraria and travel support from PCM Scientific, OPEN Health and Indivior to contribute to scientific and educational meetings. R.V. declares an honorarium and travel support from Camurus to contribute to an educational meeting. All other authors have no interests to declare.

## Data Availability

Research data are not shared.
